# Supplementary material for: Expression of Hemangioblast Proteins in von Hippel-Lindau Disease Related Tumors
Source: Cancers (Basel). 2023 Apr 29;15(9):2551. doi: 10.3390/cancers15092551 (PMC10177177; doi:10.3390/cancers15092551)
Supplement: Supplementary file 1 [file cancers-15-02551-s001.zip › cancers-2338457-supplementary.pdf]

cancers

MDPI

Supplementary Materials: Expression of Hemangioblast Proteins in von Hippel-Lindau Disease Related Tumors

Supplementary Table S1: nuclear and cytoplasmic Brachyury staining in VHL hemangioblastomas

**signal intensity**	**0**	**1**	**2**	**3**
**nuclear staining**				
CEREBELLAR	**23/26**	**0/26**	**0/26**	**3/26**
0% cells	0	0	0	0
< 10% cells	0	0	0	3
10-50% cells	0	0	0	0
50-80% cells	0	0	0	0
> 80% cells	23	0	0	0
	median Remelle score 0, mean Remelle score 0.33			
SPINAL	**11/26**	**0/21**	**1/21**	**9/21**
0% cells	0	0	0	0
< 10% cells	0	0	1	4
10-50% cells	0	0	0	3
50-80% cells	0	0	0	1
> 80% cells	11	0	0	1
	median Remelle score 1, mean Remelle score 2.65			
**cytoplasmic staining**				
CEREBELLAR	**21/26**	**4/26**	**1/26**	**0/26**
0% cells	0	0	0	0
< 10% cells	0	1	0	0
10-50% cells	0	1	0	0
50-80% cells	0	2	0	0
> 80% cells	21	0	1	0
	median Remelle score 0, mean Remelle score 0.63			
SPINAL	**18/21**	**2/21**	**1/21**	**0/21**
0% cells	0	0	0	0
< 10% cells	0	0	0	0
10-50% cells	0	1	0	0
50-80% cells	0	1	1	0
> 80% cells	18	0	0	0
	median Remelle score 0, mean Remelle score 0.55			

Supplementary Table S2: nuclear and cytoplasmic TAL1 staining in VHL hemangioblastomas

**signal intensity**	**0**	**1**	**2**	**3**
**nuclear staining**				
CEREBELLAR	**2/26**	**0/26**	**12/26**	**12/26**
0% cells	0	0	0	0
< 10% cells	0	0	2	0
10-50% cells	0	0	7	7
50-80% cells	0	0	3	4
> 80% cells	2	0	0	1

median Remelle score 6, mean Remelle score 5.08

SPINAL	**2/21**	**1/21**	**11/21**	**7/21**
0% cells	0	0	0	0
< 10% cells	0	1	3	1
10-50% cells	0	0	5	3
50-80% cells	0	0	3	2
> 80% cells	2	0	0	1

median Remelle score 4, mean Remelle score 4.67

cytoplasmic staining

CEREBELLAR	**5/26**	**1/26**	**20/26**	**0/26**
0% cells	0	0	0	0
< 10% cells	0	0	0	0
10-50% cells	0	0	6	0
50-80% cells	0	1	11	0
> 80% cells	5	0	3	0

median Remelle score 6, mean Remelle score 4.48

SPINAL	**2/21**	**6/21**	**13/21**	**0/21**
0% cells	0	0	0	0
< 10% cells	0	1	0	0
10-50% cells	0	4	4	0
50-80% cells	0	1	7	0
> 80% cells	2	0	2	0

median Remelle score 4, mean Remelle score 4.1

Supplementary Table S3: nuclear and cytoplasmic Brachyury staining in VHL clear cell renal cell carcinomas

**signal intensity**	**0**	**1**	**2**	**3**
**nuclear staining**	**11/13**	**1/13**	**1/13**	**0/13**
0% cells	0	0	0	0
< 10% cells	0	0	1	0
10-50% cells	0	1	0	0
50-80% cells	0	0	0	0
> 80% cells	11	0	0	0

median Remelle score 0, mean Remelle score 0.31

**cytoplasmic staining**	**10/13**	**3/13**	**0/13**	**0/13**
0% cells	0	0	0	0
< 10% cells	0	2	0	0
10-50% cells	0	1	0	0
50-80% cells	0	0	0	0
> 80% cells	10	0	0	0

median Remelle score 0, mean Remelle score 0.31

Supplementary Table S4: nuclear and cytoplasmic TAL1 staining in VHL clear cell renal cell carcinomas

**signal intensity**	**0**	**1**	**2**	**3**
**nuclear staining**	**1/13**	**0/13**	**6/13**	**6/13**
0% cells	0	0	0	0

< 10% cells	0	0	4	0
10-50% cells	0	0	1	2
50-80% cells	0	0	1	1
> 80% cells	1	0	0	3
	median Remelle score 2, mean Remelle score 5			
**cytoplasmic staining**	**10/13**	**3/13**	**0/13**	**0/13**
0% cells	0	0	0	0
< 10% cells	0	2	0	0
10-50% cells	0	1	0	0
50-80% cells	0	0	0	0
> 80% cells	10	0	0	0
	median Remelle score 2, mean Remelle score 2.75			

Supplementary Table S5: nuclear and cytoplasmic Brachyury staining in VHL pheochromocytomas

**signal intensity**	**0**	**1**	**2**	**3**
**nuclear staining**	**8/8**	**0/8**	**0/8**	**0/8**
0% cells	0	0	0	0
< 10% cells	0	0	0	0
10-50% cells	0	0	0	0
50-80% cells	0	0	0	0
> 80% cells	8	0	0	0
	median Remelle score 0, mean Remelle score 0			
**cytoplasmic staining**	**5/5**	**2/8**	**1/8**	**0/8**
0% cells	0	0	0	0
< 10% cells	0	0	1	0
10-50% cells	0	1	0	0
50-80% cells	0	0	0	0
> 80% cells	5	1	0	0
	median Remelle score 2, mean Remelle score 2			

Supplementary Table S6: nuclear and cytoplasmic TAL1 staining in VHL pheochromocytomas

**signal intensity**	**0**	**1**	**2**	**3**
**nuclear staining**	**5/8**	**0/8**	**2/8**	**1/8**
0% cells	0	0	0	0
< 10% cells	0	0	1	1
10-50% cells	0	0	1	0
50-80% cells	0	0	0	0
> 80% cells	5	0	0	0
	median Remelle score 0, mean Remelle score 0.86			
**cytoplasmic staining**	**1/8**	**3/8**	**4/8**	**0/8**
0% cells	0	0	0	0
< 10% cells	0	2	0	0
10-50% cells	0	0	2	0
50-80% cells	0	0	0	0
> 80% cells	1	1	2	0
	median Remelle score 3, mean Remelle score 3.25			

Supplementary Table S7: nuclear and cytoplasmic Brachyury staining in VHL pancreatic neuroendocrine tumours

**signal intensity**	**0**	**1**	**2**	**3**
**nuclear staining**	**3/5**	**0/5**	**0/5**	**2/5**
0% cells	0	0	0	0
< 10% cells	0	0	0	2
10-50% cells	0	0	0	0
50-80% cells	0	0	0	0
> 80% cells	0	0	0	0
median Remelle score 0, mean Remelle score 1.2				
**cytoplasmic staining**	**4/5**	**1/5**	**0/5**	**0/5**
0% cells	0	0	0	0
< 10% cells	0	1	0	0
10-50% cells	0	0	0	0
50-80% cells	0	0	0	0
> 80% cells	0	0	0	0
median Remelle score 0, mean Remelle score 0.2				

Supplementary Table S8: nuclear and cytoplasmic TAL1 staining in VHL pancreatic neuroendocrine tumours

**signal intensity**	**0**	**1**	**2**	**3**
**nuclear staining**	**2/5**	**0/5**	**3/5**	**0/5**
0% cells	0	0	0	0
< 10% cells	0	0	2	0
10-50% cells	0	0	1	0
50-80% cells	0	0	0	0
> 80% cells	2	0	0	0
median Remelle score 2, mean Remelle score 1.6				
**cytoplasmic staining**	**1/5**	**3/5**	**1/5**	**0/5**
0% cells	0	0	0	0
< 10% cells	0	0	0	0
10-50% cells	0	1	0	0
50-80% cells	0	0	1	0
> 80% cells	1	2	0	0
median Remelle score 4, mean Remelle score 3.2				

Supplementary Table S9: nuclear and cytoplasmic Brachyury staining in VHL paragangliomas

**signal intensity**	**0**	**1**	**2**	**3**
**nuclear staining**	**1/2**	**0/2**	**0/2**	**1/2**
0% cells	0	0	0	0
< 10% cells	0	0	0	1
10-50% cells	0	0	0	0
50-80% cells	0	0	0	0
> 80% cells	1	0	0	0
median Remelle score 2, mean Remelle score 1.5				
**cytoplasmic staining**	**1/2**	**1/2**	**0/2**	**0/2**
0% cells	0	0	0	0

< 10% cells	0	1	0	0
10-50% cells	0	0	0	0
50-80% cells	0	0	0	0
> 80% cells	1	0	0	0
	median Remelle score 1, mean Remelle score 0.5			

Supplementary Table S10: nuclear and cytoplasmic TAL1 staining in VHL paragangliomas

**signal intensity**	**0**	**1**	**2**	**3**
**nuclear staining**	**1/2**	**0/2**	**0/2**	**1/2**
0% cells	0	0	0	0
< 10% cells	0	0	0	0
10-50% cells	0	0	0	1
50-80% cells	0	0	0	0
> 80% cells	1	0	0	0
	median Remelle score 3, mean Remelle score 3			
**cytoplasmic staining**	**0/2**	**0/2**	**2/2**	**0/2**
0% cells	0	0	0	0
< 10% cells	0	0	0	0
10-50% cells	0	0	0	0
50-80% cells	0	0	0	0
> 80% cells	0	0	2	0
	median Remelle score 8, mean Remelle score 8			
